# Identification of latent biomarkers in connection with progression and prognosis in oral cancer by comprehensive bioinformatics analysis

**DOI:** 10.1186/s12957-021-02360-w

**Published:** 2021-08-12

**Authors:** Abdusemer Reyimu, Ying Chen, Xudong Song, Wubi Zhou, Jingjing Dai, Feng Jiang

**Affiliations:** 1 Medical College, Anhui University of Science and Technology, Huainan, Anhui 232001 People’s Republic of China; 2 Department of Medical Laboratory, The Affiliated Huaian No. 1 People’s Hospital of Nanjing Medical University, Huai’an, Jiangsu 223300 People’s Republic of China; 3 Department of Gastrointestinal Surgery, The Affiliated Huaian No. 1 People’s Hospital of Nanjing Medical University, Huai’an, Jiangsu 223300 People’s Republic of China; 4 Department of Pathology, The Affiliated Huaian No. 1 People’s Hospital of Nanjing Medical University, Huai’an, Jiangsu 223300 People’s Republic of China; 5 Department of Stomatology, The Affiliated Huaian No. 1 People’s Hospital of Nanjing Medical University, Huai’an, Jiangsu 223300 People’s Republic of China

**Keywords:** Oral cancer, Bioinformatics analysis, Prognosis, Central gene, Biomarkers

## Abstract

**Background:**

Oral cancer (OC) is a common and dangerous malignant tumor with a low survival rate. However, the micro level mechanism has not been explained in detail.

**Methods:**

Gene and miRNA expression micro array data were extracted from the Gene Expression Omnibus (GEO) database. The differentially expressed genes (DEGs) and miRNAs (DE miRNAs) were identified by R software. Gene Ontology (GO) enrichment and Kyoto Encyclopedia of genes and genomes (KEGG) pathway analysis were used to assess the potential molecular mechanisms of DEGs. Cytoscape software was utilized to construct protein–protein interaction (PPI) network and miRNA-gene network. Central genes were screened out with the participation of gene degree, molecular complex detection (MCODE) plugin, and miRNA-gene network. Then, the identified genes were checked by The Cancer Genome Atlas (TCGA) gene expression profile, Kaplan-Meier data, Oncomine, and the Human Protein Atlas database. Receiver operating characteristic (ROC) curve was drawn to predict the diagnostic efficiency of crucial gene level in normal and tumor tissues. Univariate and multivariate Cox regression were used to analyze the effect of dominant genes and clinical characteristics on the overall survival rate of OC patients.

**Results:**

Gene expression data of gene expression profiling chip(GSE9844, GSE30784, and GSE74530) were obtained from GEO database, including 199 tumor and 63 non-tumor samples. We identified 298 gene mutations, including 200 upregulated and 98 downregulated genes. GO functional annotation analysis showed that DEGs were enriched in extracellular structure and extracellular matrix containing collagen. In addition, KEGG pathway enrichment analysis demonstrated that the DEGs were significantly enriched in IL-17 signaling pathway and PI3K-Akt signaling pathway. Then, we detected three most relevant modules in PPI network. Central genes (CXCL8, DDX60, EIF2AK2, GBP1, IFI44, IFI44L, IFIT1, IL6, MMP9,CXCL1, CCL20, RSAD2, and RTP4) were screened out with the participation of MCODE plugin, gene degree, and miRNA-gene network. TCGA gene expression profile and Kaplan-Meier analysis showed that high expression of CXCL8, DDX60, IL6, and RTP4 was associated with poor prognosis in OC patients, while patients with high expression of IFI44L and RSAD2 had a better prognosis. The elevated expression of CXCL8, DDX60, IFI44L, RSAD2, and RTP44 in OC was verified by using Oncomine database. ROC curve showed that the mRNA levels of these five genes had a helpful diagnostic effect on tumor tissue. The Human Protein Atlas database showed that the protein expressions of DDX60, IFI44L, RSAD2, and RTP44 in tumor tissues were higher than those in normal tissues. Finally, univariate and multivariate Cox regression showed that DDX60, IFI44L, RSAD2, and RTP44 were independent prognostic indicators of OC.

**Conclusion:**

This study revealed the potential biomarkers and relevant pathways of OC from publicly available GEO database, and provided a theoretical basis for elucidating the diagnosis, treatment, and prognosis of OC.

## Introduction

Oral cancer (OC) is part of the most common types of head and neck squamous cell carcinoma (HNSCC), which is highly invasive and prone to local recurrence and metastasis [1]. The most common type of OC is oral squamous cell carcinoma (OSCC), accounting for more than 90% of OC [2]. The treatment effect and prognosis of OC patients are indigent, and the 5-year survival rate is about 50% [3]. Although significant progress has been made in OC treatment, the 5-year survival rate of OC individuals is still very low [4]. With the rapid development of micro array technology, micro-array technology has been widely utilized to obtain and study the gene expression profile data of human cancer [5]. It also provides a theoretical basis for exploring the relationship between gene expression and regulation in life science [6].

In this project, we download the data of mRNA expression micro array (GSE9844, GSE30784, and GSE74530) and miRNA expression micro array (GSE124566) from Gene Expression Omnibus(GEO) public database. We use limma package in Rstudio to process the data and screen out the DEGs and DE miRNAs. Gene Ontology (GO) function annotation and Kyoto Encyclopedia of genes and genomes (KEGG) path analysis of differentially expressed genes (DEGs) are carried out through clusterprofiler package. In addition, PPI network [7] and miRNA-gene network [8] were built and key genes were screened. The Kaplan-Meier plotter was used for overall survival analysis to further verify the core genes.

Based on these above-mentioned findings, it is highly conceivable that it provides a theoretical basis and reference for the study of pathological mechanism, prevention, early diagnosis, and drug target therapy of OC.

## Materials and methods

### Expression profile data collection

Three mRNA expression datasets (GSE9844, GSE30784, and GSE74530) and one miRNA expression dataset (GSE124566) were downloaded from the GEO database (https://www.ncbi.nlm.nih.gov/geo/). The mRNA datasets were inspired by GPL570 platform (Affymetrix Human Genome U133 Plus 2.0 Array) (Affymetrix, Santa Clara, CA, USA). GSE9844 dataset includes 26 tumor samples and 12 control samples, GSE30784 dataset includes 167 tumor samples and 45 control samples, and GSE74530 dataset includes 6 tumor tissues and 6 precancerous tissues. In addition, GSE124566 dataset is built on GPL18402 platform, including 10 tumor tissues and 10 corresponding normal tissues.

### DEGs identification

The original data was normalized by RMA algorithm in Affy package of R software. The probe ID was converted to gene name by GPL570 platform, and limma package was used for DEGs analysis [9]. DEGs were further identified according to |log_2_FC| > 1 and *P* < 0.05. The upregulated or downregulated DEGs in the data set were integrated by Venn analysis.

### GO and KEGG enrichment analyses of DEGs

GO and pathway annotation and enrichment analyses were based on the NCBI COG (http://www.ncbi.nlm.nih.gov/COG/), Gene Ontology Database (http://www.geneontology.org/) and KEGG pathway Database (http://www.genome.jp/kegg/), respectively. Clusterprofiler is an R software package which supports GO and KEGG enrichment and visualization of analysis results. In this study, clusterprofiler software package was used to analyze the GO and KEGG path enrichment of DEGs. *P* < 0.05 was identified as the cut-off standard of significant enrichment.

### PPI network construction and module analysis

STRING (http://www.string-db.org/) was a commonly used online search tool to predict PPI network of candidate genes at protein level [10]. In PPI network of DEGs, the interaction score ≥ 0.4 was used as the screening condition. Cytoscape software (version 3.8.2) was utilized to visualize PPI network. MCODE plugin was utilized to analyze PPI network function module [11]. The module filtering parameters were set as node score cutoff = 0.2, k-core = 2, max. depth = 100 and degree cutoff = 2. DAVID (https://david.ncifcrf.gov/) online tool was used for enrichment analysis of critical module genes.

### Establishment of miRNA-gene network

We selected the bidirectional search function in mirDIP database (http://ophid.utoronto.ca/mirDIP/index_confirm.jsp) to predict the bidirectional relationship between DE miRNAs and DEGs. Mirtar, Mirdb, and Targetscan are selected to predict. The 5% genes with the highest confidence level were considered as potential target genes. The miRNA-gene network was visualized by Cytoscape software. In order to identify the hub gene, we combined the results of MCODE, degree, and miRNA-gene network.

### Verification of OC hub genes

In checking to see the hub genes in OC, we tried to verify the central genes through TCGA dataset. A total of 305 samples were included, including 274 OC tissues and 31 adjacent non tumor tissues. The expression level of each dominant gene was extracted for further analysis. The Student’s *t* test was used to statistically analyze the crucial genes between OC and adjacent non-tumor tissues. The prognosis of patients was evaluated by Kaplan-Meier analysis. The difference of logarithmic rank test was statistically significant, and the selected genes were further verified by Oncomine database. In addition, according to the Human Protein Atlas database (http://www.proteinatlas.org/), the expression levels of 6 genes selected from OC and normal tissues were analyzed by immunohistochemistry(IHC). Finally, ROC curve analysis of the ability of principal genes to distinguish OC from normal tissues. Univariate and multivariate Cox regression analysis was conducted to determine the hub genes related to the prognosis of OC patients.

## Results

### Identification of DEGs in OC

**Fig. 1:**
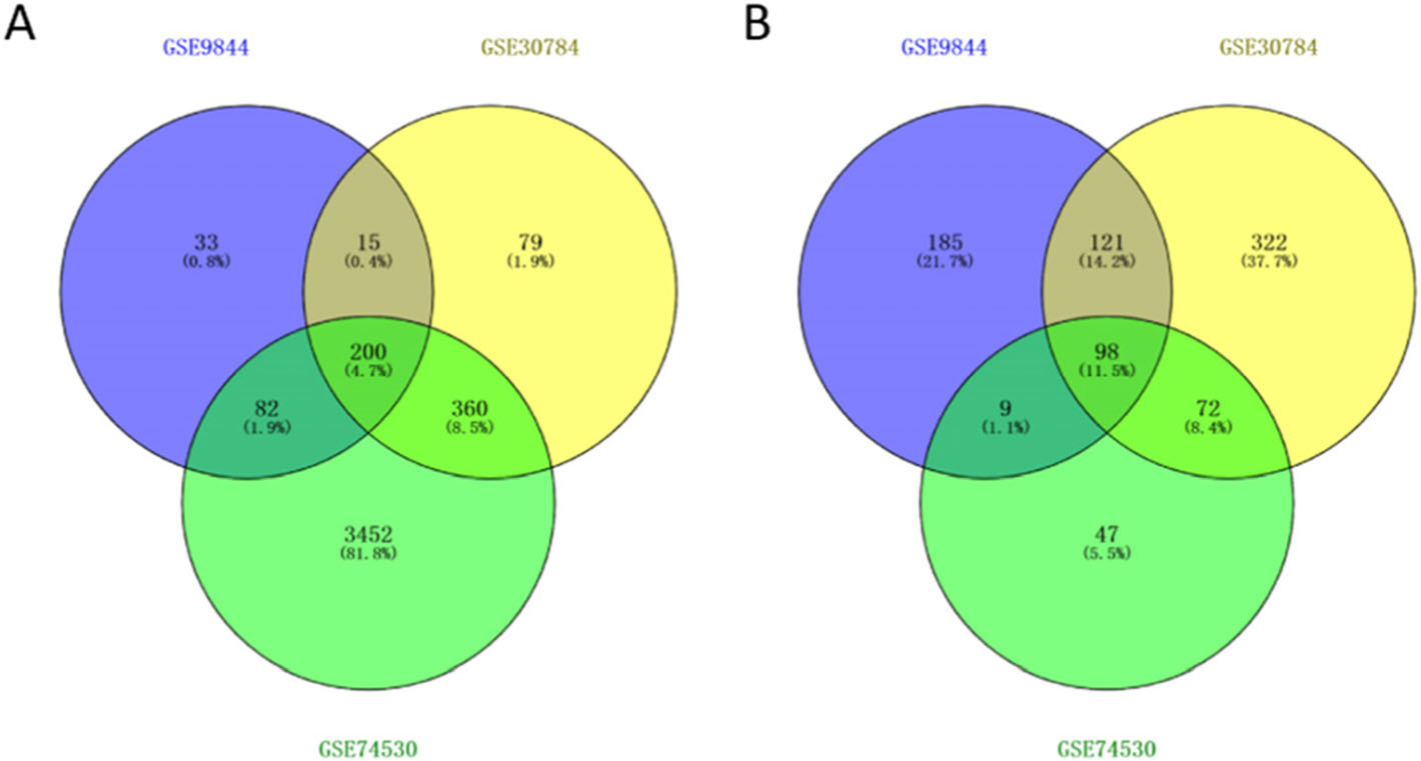
Recognition of overlapping DEGs. **A** Venn map of 200 overlapping upregulated genes in GSE9844, GSE30784, and GSE74530. **B** Venn map of 98 overlapping downregulated genes in the same datasets were identified

Three datasets were collected from the GEO database, including OC tissue samples and normal oral tissue samples: GSE9844, GSE30784, and GSE74530. The R package of “limma” was used for analysis, and screening conditions were adjusted *P* < 0.05, |log_2_FC| > 1. Compared with normal oral tissue samples, 298 genes were obtained in OC tissue samples, including 200 upregulated genes and 98 downregulated genes (Fig. 1).

### Function and pathway enrichment of DEGs

**Fig. 2:**
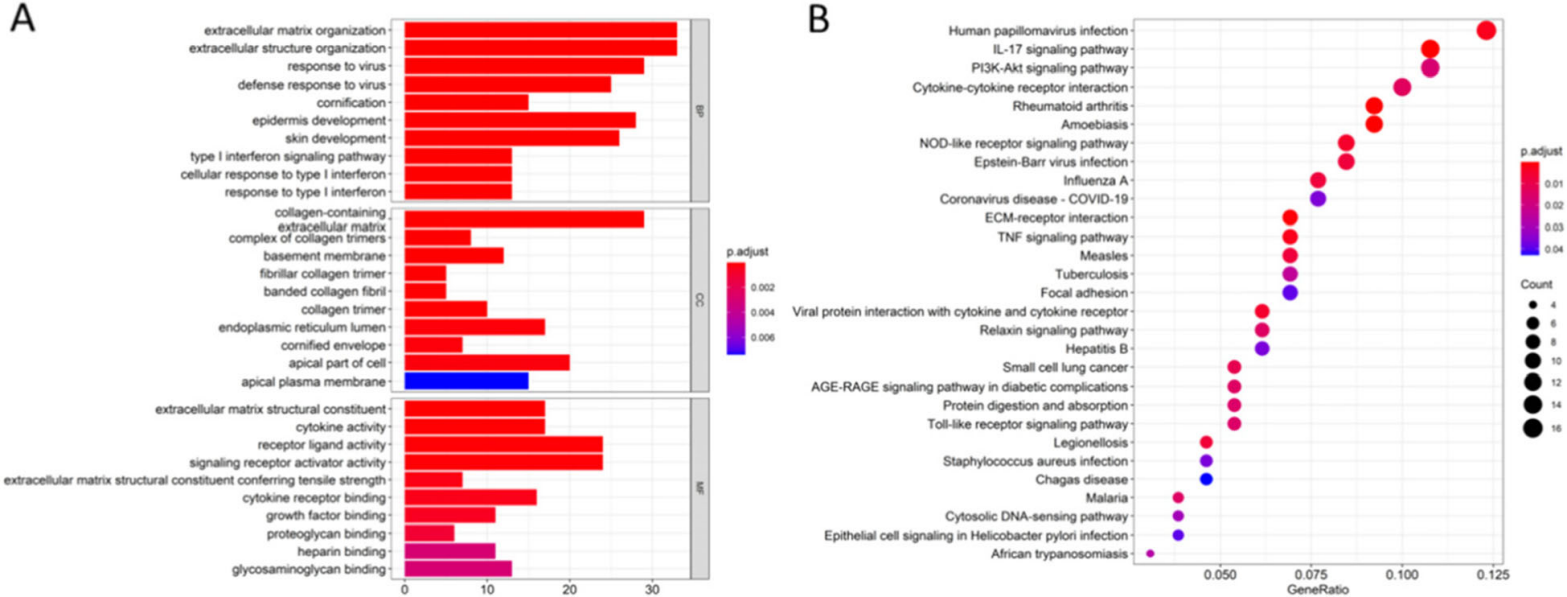
Clusterprofiler package was used for enrichment analysis of DEGs by GO term and KEGG pathways

GO functional annotation analysis revealed that 298 overlapping genes were involved in many biological process (BP), including extracellular structure organization, response to virus, defense response to virus cornification, epidermis development, skin development, type l interferon signaling pathway, and response to type l interferon (Fig. 2A). For cellular component (CC), DEGs was mainly distributed in collagen-containing extracellular matrix, complex of collagen trimers, basement membrane, fibrillar collagen trimer, banded collagen fibril, collagen trimer, endoparasitic reticulum lumen, certified envelope, apical part of cell, and apical plasma membrane (Fig. 2A). Overlapping DEGs were mainly related to extracellular matrix structural constituent, cytokine activity, receptor ligand activity, signaling receptor activator activity, extracellular matrix structural constituent conferring tensile strength, cytokine receptor binding, growth factor binding, proteoglycan binding, heparin binding, and glycosaminoglycan binding in terms of molecular function (MF) (Fig. 2A). Furthermore, the KEGG pathways of DEGs included IL-17 signaling pathway, PI3K-Akt signaling pathway, cytokine–cytokine receptor interaction, NOD-like receptor signaling pathway, ECM-receptor interaction, and TNF signaling pathway (Fig. 2B).

### PPI network and modules analysis

**Fig. 3:**
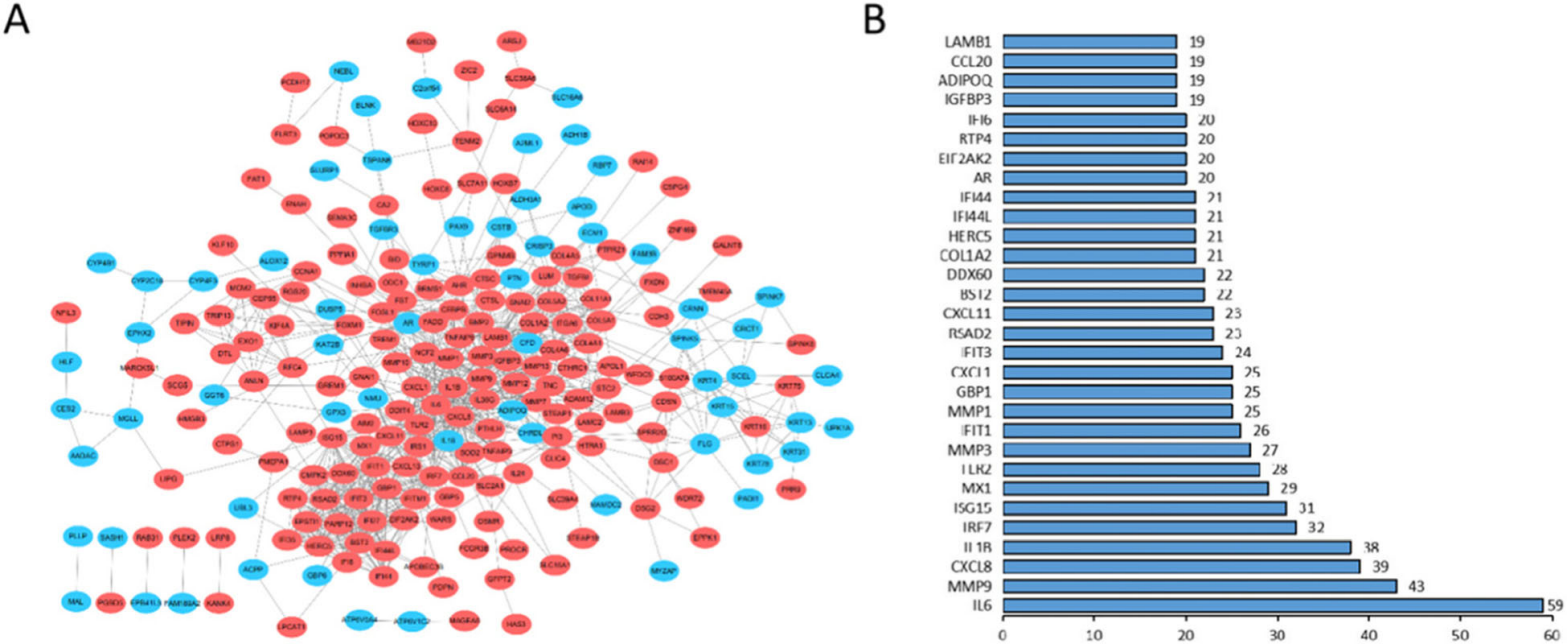
DEGs network. **A** 229 DEGs were filtered in string online database and PPI network was visualized by Cytoscape. **B** Top 30 nodes in PPI network according to degree.

**Fig. 4:**
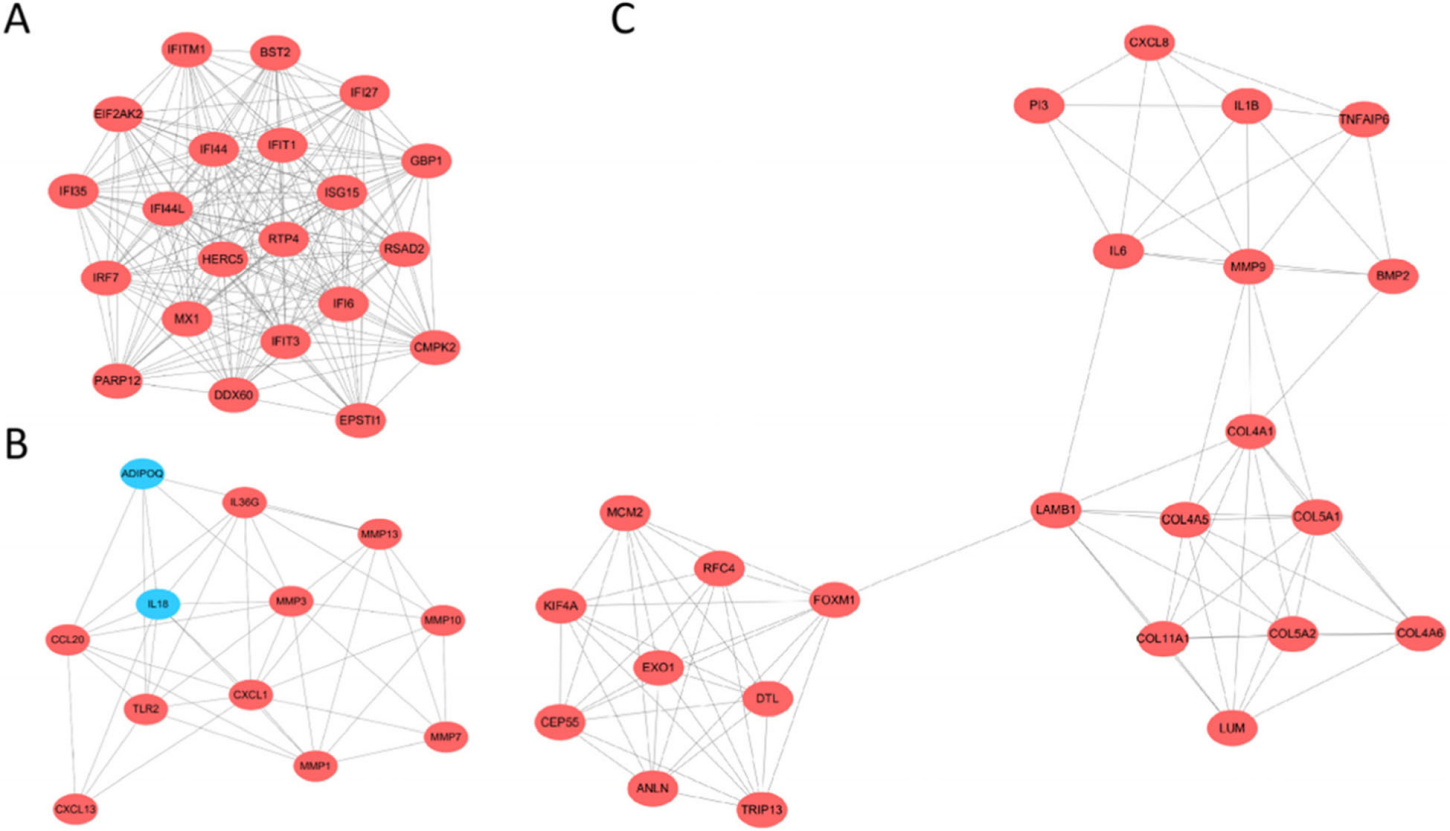
Selecting the most important modules from the PPI network. **A** Module 1. **B** Module 2. **C** Module 3

**Fig. 5:**
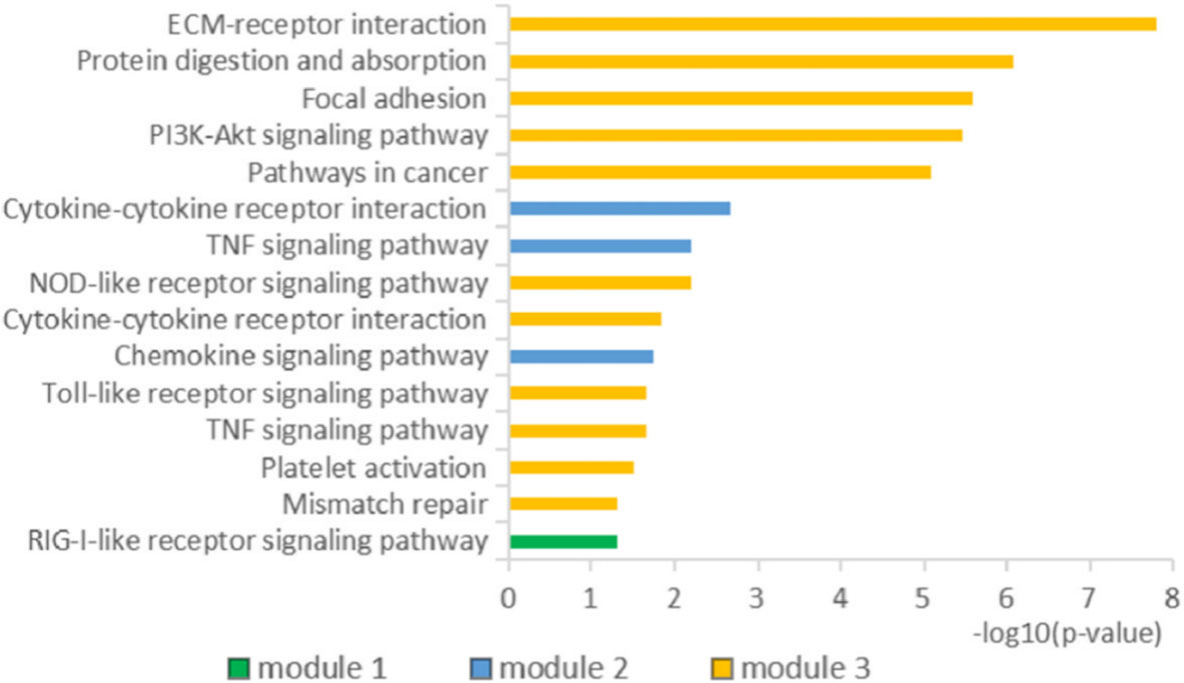
Enrichment analysis of KEGG pathway of three module genes. The length of bar indicates the enrichment degree of module gene

STRING database involved 229 DEGs into PPI network, showed 229 nodes and 925 edges (Fig. 3A), and identified 30 essential proteins (Fig. 3B). Then, we used MCODE plug-in to obtain three modules. Among them, module 1 contains 21 core proteins with the highest score (Fig. 4A), module 2 contains 12 proteins (Fig. 4B), and module 3 contains 24 proteins (Fig. 4C). It may indicate that 57 DEGs have a significant impact on OC. Moreover, the enrichment analysis of KEGG pathway showed that the module genes were enriched in ECM-receptor interaction, protein digestion and absorption, focal adhesion, PI3K-Akt signaling pathway, pathways in cancer, cytokine–cytokine receptor interaction, TNF signaling pathway, NOD-like receptor signaling pathway, chemokine signaling pathway, Toll-like receptor signaling pathway, platelet activation, mismatch repair, and RIG-I-like receptor signaling pathway (Fig. 5).

### Construction of the miRNA-gene network

**Fig. 6:**
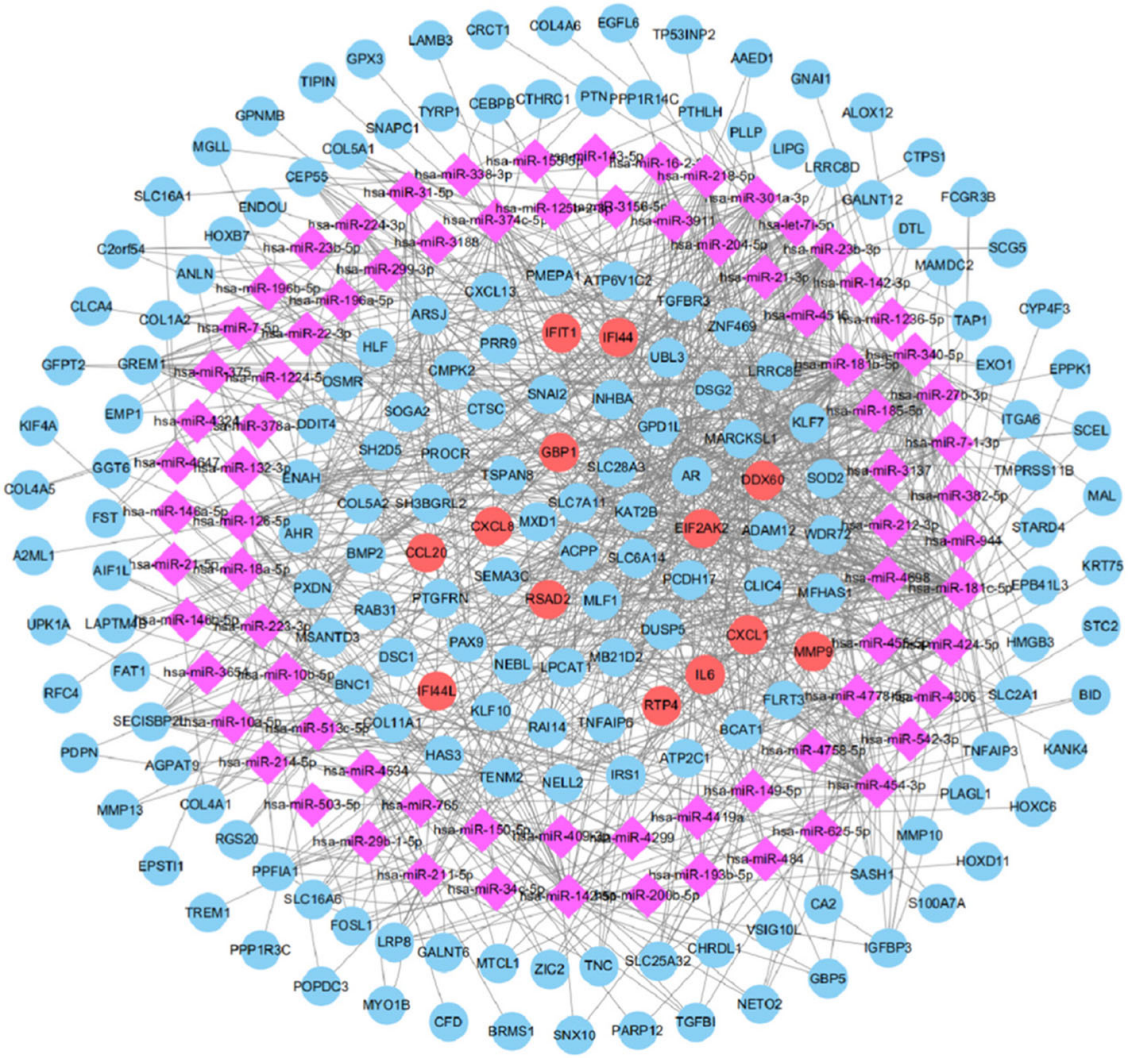
The regulatory network of miRNA gene and 13 hub genes. Round nodes represent DEGs, red round nodes represent 13 hub genes, and purple diamond nodes stand for DE miRNAs

A total of 103 DE-miRNAs were screened from the miRNA dataset (GSE124566). In addition, three programs (miRTar, miRDB, and TargetScan) were utilized to screen the target genes of DE-miRNAs. In addition, in order to identify credible hub genes, DE-miRNAs target genes were compared with DEGs, and overlapping genes were used as hub genes. Combined with the results of MCODE analysis, degree and miRNA-gene network, 13 hub genes were selected, which were upregulated DEGs, including CXCL8, DDX60, EIF2AK2, GBP1, IFI44, IFI44L, IFIT1, IL6, MMP9,CXCL1, CCL20, RSAD2, and RTP4 (Fig. 6).

### Verification of hub gene

**Fig. 7:**
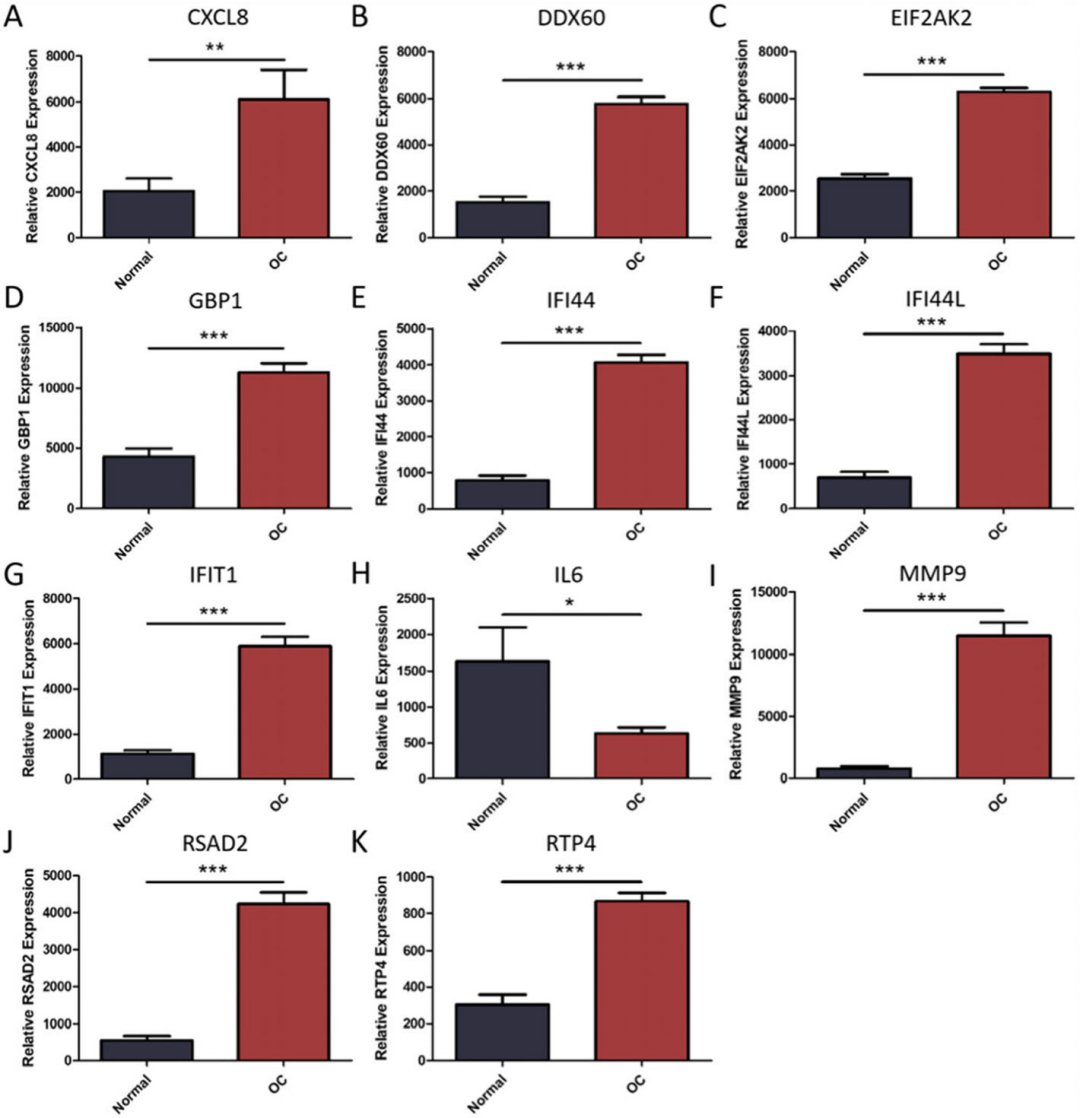
Using TCGA database to verify the difference of hub gene expression between OC and non-tumor tissues. These points represent the level of gene expression. **A** CXCL8. **B** DDX60. **C** EIF2AK2. **D** GBP1. **E** IFI44. **F** IFI44L. **G** IFIT1. **H** IL6. **I** MMP9. **J** RSAD2. **K** RTP4. The red box was OC (oral cancer), the gray box was normal tissue, *P < 0.05, * *P < 0.01, * *P < 0.001

**Fig. 8:**
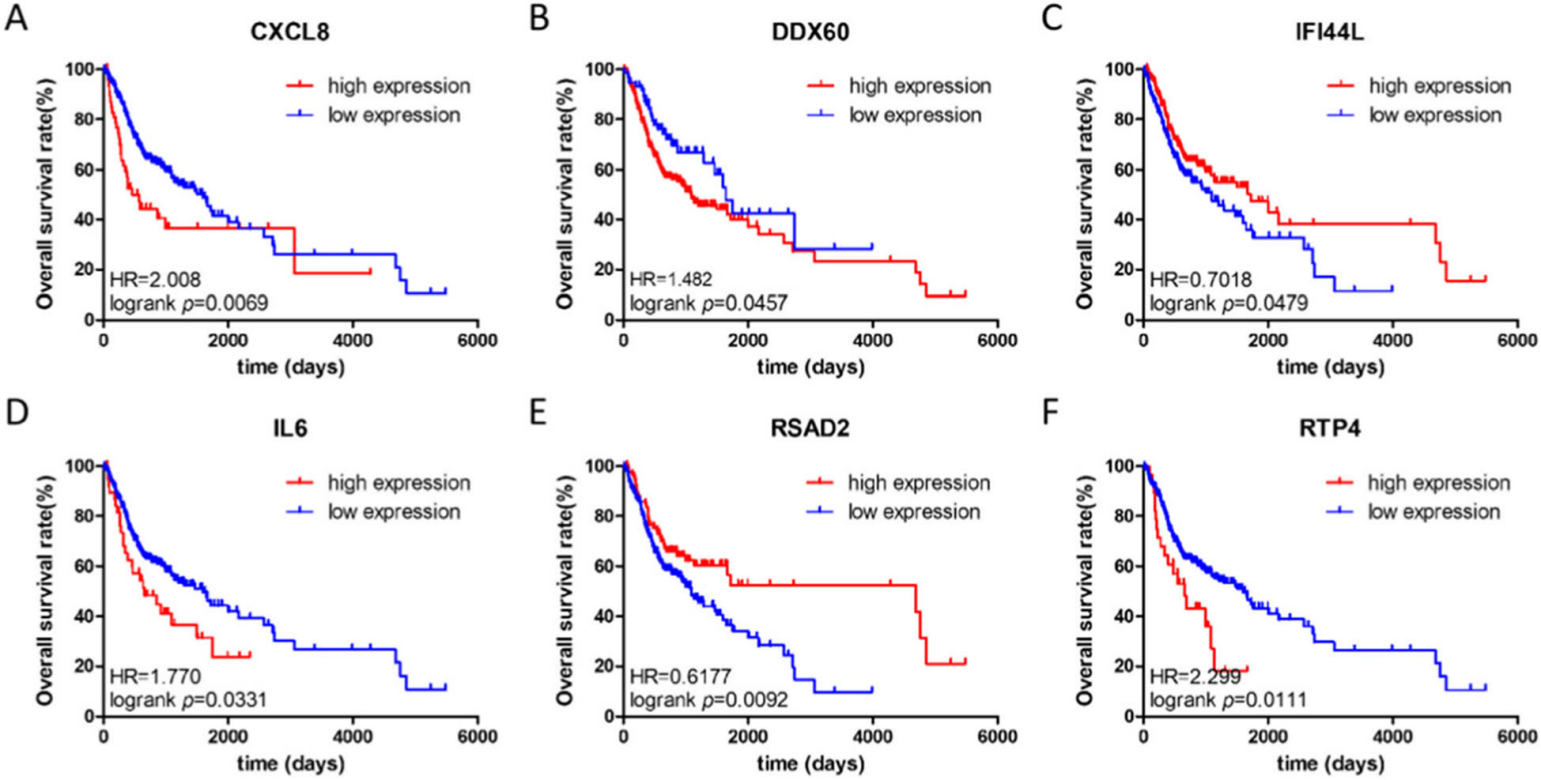
The overall survival rate (OS) curve of 6 hub genes. **a** CXCL8. **b** DDX60. **c** IFI44L. **d** IL6. **e** RSAD2. **f** RTP4

**Fig. 9:**
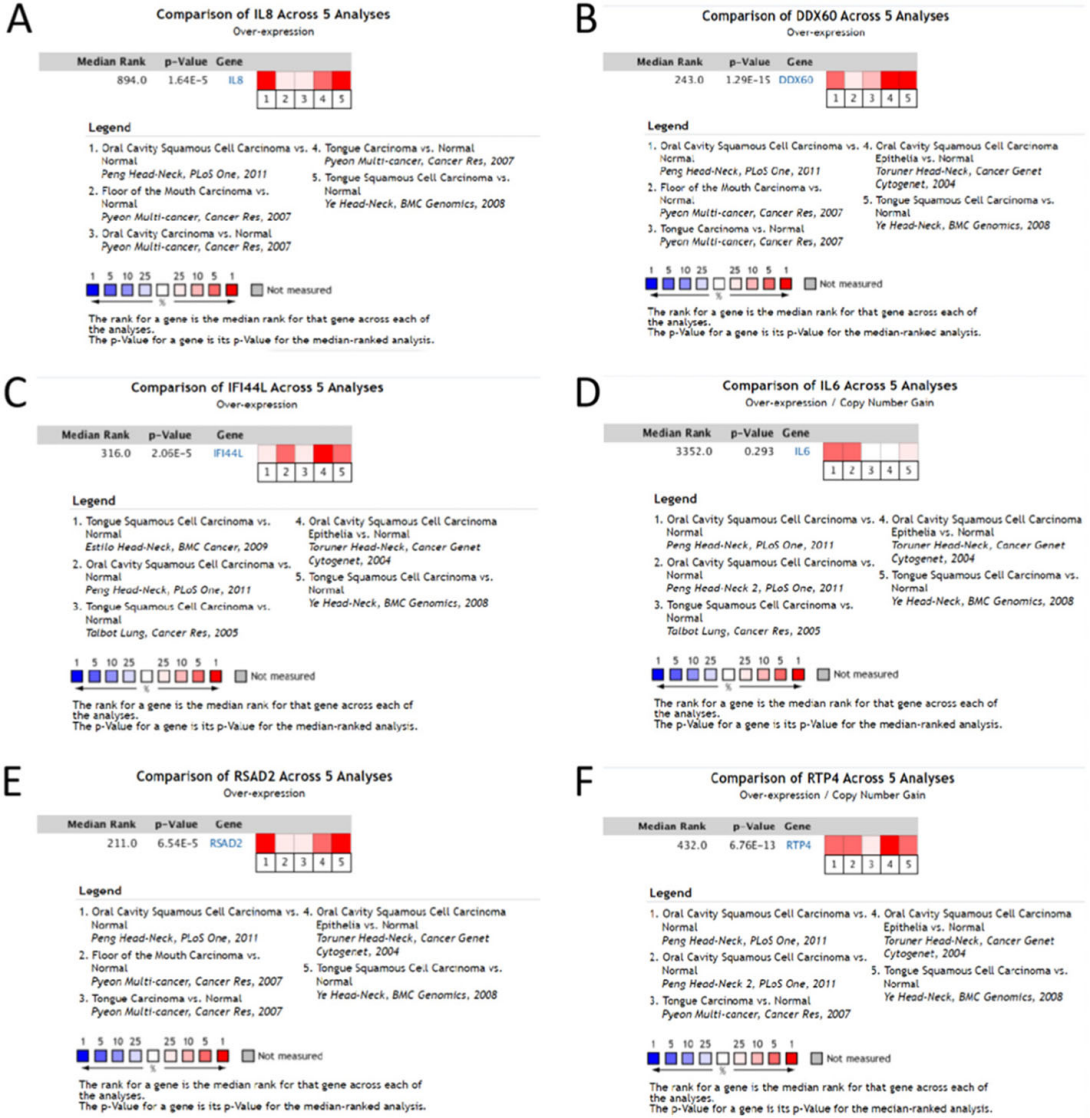
Oncomine database showed the expression of 6 hub genes in multiple OC datasets. **a** CXCL8. **b** DDX60. **c** IFI44L. **d** IL6. **e** RSAD2. **f** RTP4

**Fig. 10:**
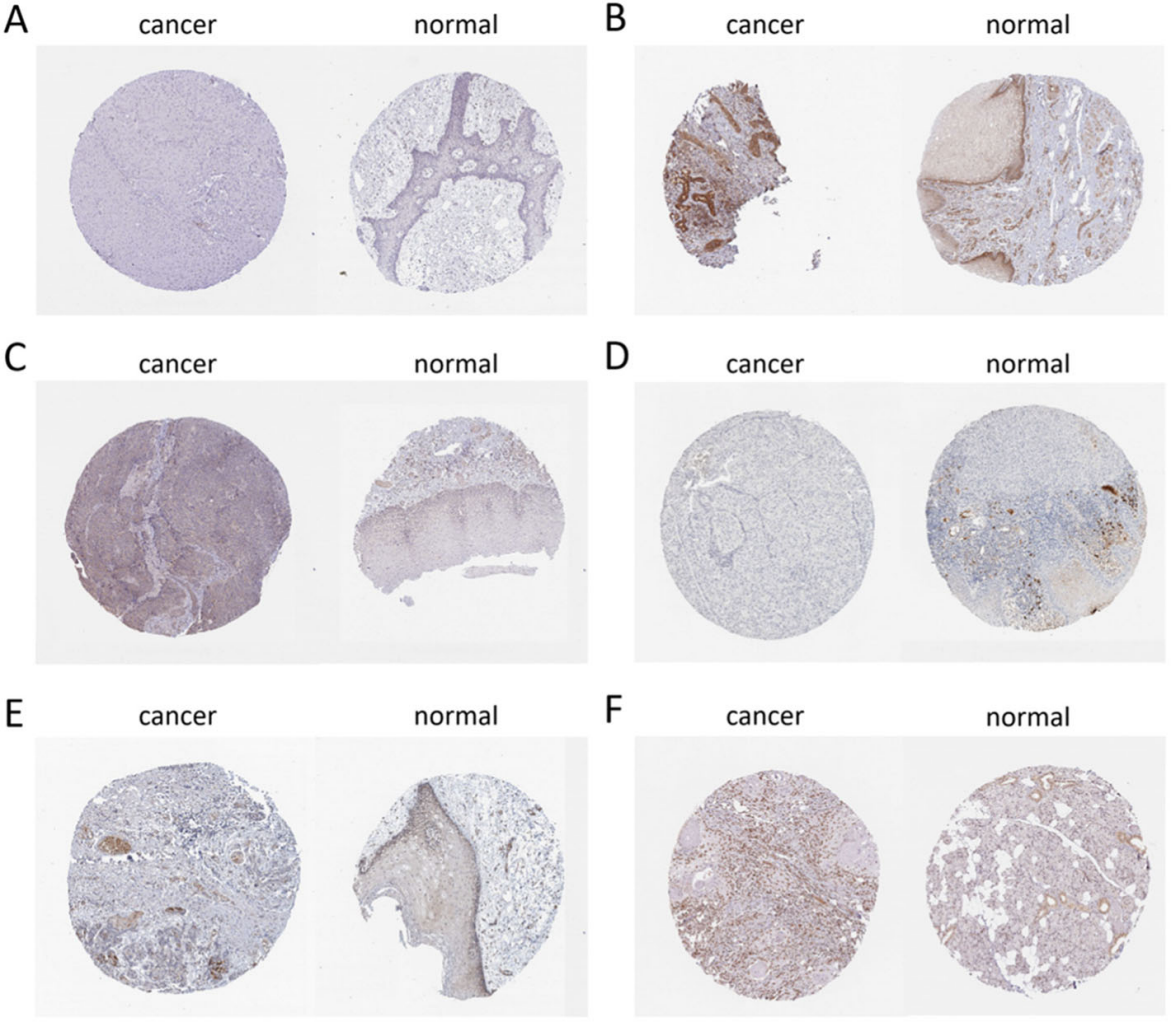
Immunohistochemistry of two key genes based on the Human Protein Atlas. **A** Protein levels of CXCL8 in tumor tissue (staining: Not detected; intensity: Negative; quantity: None). Protein levels of CXCL8 in normal tissue (staining: not detected; intensity: weak; quantity: < 25%). **B** Protein levels of DDX60 in tumor tissue (staining: high; intensity: strong; quantity: > 75%). Protein levels of DDX60 in normal tissue (staining: low; intensity: moderate; quantity: < 25%). **C** Protein levels of IFI44L in tumor tissue (staining: low; intensity: weak; quantity: 75–25%). Protein levels of IFI44L in normal tissue (staining: not detected; intensity: weak; quantity: < 25%). **D** Protein levels of IL6 in tumor tissue (staining: not detected; intensity: negative; quantity: None). Protein levels of IL6 in normal tissue (staining: low; intensity: moderate; quantity: < 25%). **E** Protein levels of RSAD2 in tumor tissue (staining: medium; intensity: strong; quantity: < 25%). Protein levels of RSAD2 in normal tissue (staining: low; intensity: moderate; quantity: < 25%). **F** Protein levels of RTP4 in tumor tissue (staining: high; intensity: strong; quantity: > 75%). Protein levels of RTP4 in normal tissue (staining: medium; intensity: moderate; quantity: > 75%)

**Figure 11:**
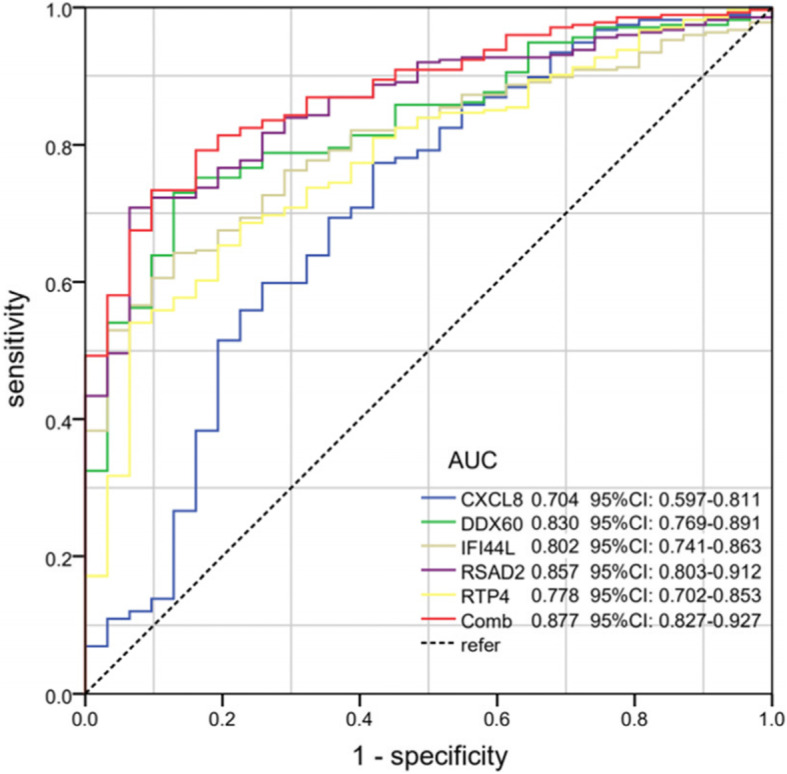
Receiver operating characteristic (ROC) curve analysis and area under curve (AUC) statistics were used to evaluate the ability of key genes to distinguish OC from normal tissues.

The selected hub was checked by TCGA database. Among them, 11 hub genes (CXCL8, DDX60, EIF2AK2, GBP1, IFI44, IFI44L, IFIT1, IL6, MMP9, RSAD2, and RTP4) were differentially expressed in OC (Fig. 7). At the same time, we found that the expression of CXCL8, DDX60, IFI44L, IL6, RSAD2, and RTP4 was substantially correlated with the survival rate of OC patients (Fig. 8). The expression analysis of OC and normal tissues in Oncomine database also showed that the expression levels of these five genes(CXCL8, DDX60, IFI44L, RSAD2, and RTP4) were higher in OC samples (Fig. 9). The Human Protein Atlas database showed that the protein expressions of DDX60, IFI44L, RSAD2, and RTP44 in tumor tissues were higher than those in normal tissues (Fig. 10). Furthermore, the ROC curve analysis showed that hub gene (CXCL8, DDX60, IFI44L, RSAD2, and RTP4) could distinguish OC from normal tissues, and the diagnosis effect of tumor tissue was better, and the combined diagnosis of five genes was the best (Fig. 11). Finally, univariate and multivariate Cox regression showed that DDX60, IFI44L, RSAD2, and RTP4 were independent prognostic indicators of overall survival in OC patients (Table 1).

**Table 1:** Univariate and multivariate analyses of the correlation of CXCL8, DDX60, IFI44L, RSAD2, and RTP4 expression with overall survival (OS) among OC patients

Variables	Univariate analysis			Multivariate analysis		
	HR	95%CI	*P*	HR	95%CI	*P*
Age (< 60 vs. > 60)	0.741	0.524–1.048	0.090	0.684	0.944–1.722	**0.040**
Stage (stages I and II vs. stages III and IV)	0.511	0.331–0.789	**0.002**	0.480	0.542–1.622	**0.001**
Grade (G1 and G2 vs. G3 and G4)	0.799	0.537–1.188	0.268	0.701	0.752–1.868	0.098
CXCL8	0.564	0.370–0.859	**0.008**	0.715	0.960–1.048	0.138
DDX60	0.647	0.420–0.995	**0.047**	0.393	0.990–1.021	**0.000**
IFI44L	1.419	1.001–2.012	**0.049**	1.739	0.885–1.134	**0.029**
RSAD2	1.689	1.133–2.512	**0.010**	1.853	0.990–1.014	**0.017**
RTP4	0.526	0.318–0.871	**0.013**	0.351	0.956–0.998	**0.000**

Bold values indicate *P* < 0.05. *HR* hazard ratio, *Cl* confidence interval

## Discussion

Although surgical treatment, radiotherapy, and chemotherapy have been developing in recent decades, the therapeutic effect and mortality of OC are still not greatly improved [12]. The risk factors for OC involve an interaction between the habits, environmental (tobacco, betelquid, alcohol, HPV, etc.), and genetic (EGFR, TP53, Her-2-neu, GADD45, VEGF, cyclin D1, HIF-1a, CDKN2A, DNA methylation, CRP, etc.) factors [12–20]. For instance, DNA methylation and abnormal gene expression lead to changes in CpG island methylation phenotype and lower survival rate [13]. EGFR, Her-2-neu, and GADD45 are closely related to the sensitivity of chemical drugs [14]. Therefore, finding possible oral cancer-related markers has become a top priority.

Nowadays, the research direction of OC is changing from looking for disease-specific genes to understand the biochemical and molecular functions of various genes and how complex interaction networks affect the occurrence, development, and prognosis of the disease. In this project, 298 DEGs (200 upregulated genes, 98 downregulated genes) were screened by downloading three mRNA micro array data from GEO public database, and further functional analysis was carried out.

In addition, the results of GO analysis showed that DEGs were mainly enriched in the extracellular matrix, type I interference pathway and collagen fiber, such as extracellular structure organization, type I interfere signaling pathway, fibrinous collagen trimer, and banded collagen fiber. In recent years, a lot of research have shown that the degradation of extracellular matrix and basement membrane plays a decisive role in the migration and invasion of cancer cells [21]. Interferon I can reverse the immunosuppressive effect of MSCs (mesenchyme stem cells) and effectively activate the immune response of the body, so as to play the role of tumor immunotherapy [22]. Now more evidence shows that collagen actively participates in tumor progression through its degradation, remodeling, changing the adhesion of tumor cells, promoting tumor angiogenesis, and basement membrane destruction [23]. In addition, KEGG pathway enrichment showed that DEGs were significantly enriched in IL-17 signaling pathway, PI3K-Akt signaling pathway, cytokine–cytokine receptor interaction, NOD-like receptor signaling pathway, ECM-receptor interaction, TNF signaling pathway, and focal adhesion. IL-17 can send signals to colorectal cancer cells and inhibit the production of CXCL9/10 chemokines. Thereby inhibiting the infiltration of CD8 CTL and Tregs into colorectal cancer and promoting the development of colorectal cancer [24]. PI3K/Akt signaling pathway promotes the growth and invasion of gastric cancer cells by preventing apoptosis [25]. Ganoderma lucidum extract regulates cytokines and stimulates anti-tumor immunostimulatory activity through cytokine–cytokine receptor interaction [26]. Nod-like receptor signaling molecules promote glioma angiogenesis by acting on downstream targets such as IL-1 and caspase-1 [27]. Mir-215-5p over expression reduces the migration and invasion of colorectal cancer cells through the interaction of extracellular matrix receptors [28]. SPRY4-IT1 promotes hepatocarcinogenesis and metastasis through TNF signaling pathway [29]. Microrna-27b inhibits tumor metastasis and adhesion by regulating the focal adhesion pathway [30]. These key pathways are related to the progress of human tumor and have certain guiding significance for the exploration of OC therapeutic targets.

PPI network analyzes DEGs and shows 298 nodes. MCODE plugin selects the most significant three related modules. The results of MCODE analysis, degree, and miRNA gene network were used to identify the central gene. In addition, we verified our results by TCGA database gene expression profile data, survival analysis, and immunohistochemistry, which improved the reliability of our results. Among these genes, CXCL8, DDX60, IFI44l, IL6, RSAD2, and RTP44 were significantly abnormal in OC and had an impact on the prognosis of patients. ROC curve analysis showed that CXCL8, DDX60, IFI44l, RSAD2, and RTP44 had better diagnostic efficiency for normal and tumor tissues, and the combined diagnosis was more effective. Meanwhile, univariate and multivariate Cox proportional hazards regression showed that DDX60, IFI44l, RSAD2, and RTP44 were independent prognostic indicators of OC.

Interleukin 8 (CXCL8/IL8) is secreted by tumor cells and acts with CXCR1/2 in tumor microenvironment to regulate the proliferation and self-renewal of cancer stem cells (CSCs), which plays an important role in tumor progression and metastasis by regulating inflammatory factors [31].Some inflammatory markers such as CXCL8, interleukin-6, tumor necrotic factor, and C-reactive protein (CRP) were recently identified as prognostic markers in oral cavity cancer [17]. Upregulation of CXCL8 stimulates the proliferation and migration of HCC cells, which is closely related to clinical stage and tumor infiltration [32]. CXCL8 secreted by tumor associated macrophages (TAM) inhibits ER + expression in endometrial carcinoma (EC) cells through HOXB13, which may be related to invasion, metastasis, and poor prognosis of cancer [33]. The results of this study suggest that over expression of CXCL8 in OC is associated with poor prognosis.

DExD/H-Box Helicase 60 (DDX60) is a kind of dead box RNA helicase, and breast cancer patients with low expression of DDX60 are more sensitive to radio [34]. The expression of DDX60 is related to the stage of cancer and has a potential key to the diagnosis, prognosis, and treatment of colorectal cancer (CRC) [35]. Similarly, our study shows that DDX60 is highly expressed at mRNA and tissue levels, and is negatively correlated with the prognosis of OC patients, which may be a potential independent indicator and therapeutic target of OC.

Interferon-induced protein 44 like (IFI44L) is a tumor suppressor gene, which regulates the Met/SRC signaling pathway in hepatocellular carcinoma to affect cancer cell migration, lung metastasis, and drug resistance, and can be used as an ideal prognostic marker [36]. Compared with normal bone cells, the expression of IFI44L in osteosarcoma was decreased, and the high expression of IFI44L indicated a good prognosis in osteosarcoma patients [37]. Consistent with previous studies, the expression of IFI44L is positively correlated with the prognosis of patients with OC, and the high expression of IFI44L may play an anti-tumor role in OC. Therefore, IFI44L is supposed to be a potential prognostic indicator of OC.

Interleukin-6 (IL-6) is a typical tumor promoting cytokine in the interleukin-6 cytokine family, which regulates various STAT3-mediated carcinogenesis [38, 39]. Paradoxically, more and more evidence shows that some interleukin-6 family cytokines can also produce anti-tumor response. For example, in a phosphatase and tension homolog (PTEN)-deficient prostate cancer model, IL-6-STAT3 signaling in tumor cells prevents tumor progression by maintaining a complete senescence-induced ARF-MDM2-p53 tumor suppressor axis [40]. Therefore, our challenge is to find the dynamic interactions between pro- and anti-tumor activities in different tissue compartments. In our study, IL-6 expression was low in OC, and the prognosis of patients with low expression was better.

Radial S-adenosyl methionine domain containing protein 2 (RSAD2) promotes the maturation of dendritic cells (DCS) through IRF7 mediated signaling pathway. However, in RSAD2 knockout mice with lung metastasis, MDCs lose their anti-tumor effect [41], which also reflects that antigen-presenting cells are crucial for inducing cancer immunity [42]. A bioinformatics study on breast cancer reported that RSAD2 expression was associated with tumor grade, stage, and size [43]. Similarly, our study shows that the high expression of RSAD2 in OC has a positive impact on the prognosis of patients. It is speculated that RSAD2 in OC may have a similar anti-tumor effect with the above studies. Its molecular mechanism needs further study in vivo and in vitro.

The expression level of receptor transporter protein 4 (RTP4) can be used to independently predict the outcome of breast cancer in HER2 (+) patients, and its high expression level is associated with poor survival and prognosis [44]. Recent studies have shown that the methylation center gene RTP4 in prostate adenocarcinoma can be regarded as a biomarker for the diagnosis and treatment of prostate cancer [45]. Consistent with previous studies, the high expression of RTP4 in OC is associated with poor prognosis, and the analysis results show that RTP4 may be an independent prognostic indicator of OC.

Our research has the following limitations. First of all, our research is based on data analysis, and the results need to be verified by in vivo and in vitro experiments. Secondly, we lack the molecular mechanism of these genes, and we will combine these genes for additional exploration. In the future, we will further design experiments (including western blot, immunohistochemistry, etc.) based on the specific mechanism, conduct in-depth research, and improve the shortcomings.

## Conclusion

In conclusion, we have performed prognostic analyses in oral cancer via a bioinformatics approach, resulting in the identification of latent prognostic hub genes with adequate reliability and sensitivity. Future investigations are warranted to validate this signature in prospective clinical trials and unravel the tumorigenic roles of these biomarkers in OC.

## Data Availability

The datasets supporting the conclusion of this article are included within the article.

## Abbreviations

OC: Oral cancer
GEO: Gene Expression Omnibus
DEGs: Differentially expressed genes
GO: Gene Ontology
KEGG: Kyoto Encyclopedia of Genes and Genomes
PPI: Protein–protein interaction
MCODE: Molecular complex detection
ROC: Receiver operating characteristic
MF: Molecular function
CC: Cell components
BP: Biological process
OS: Overall survival
MSCs: Mesenchymal stem cells
CSCs: Cancer stem cells
TAM: Tumor-associated macrophages
EC: Endometrial carcinoma
DDX60: DEXD/H box helicase 60
CRC: Colorectal cancer
IFI44L: Interferon-induced protein 44 like
HCC: Hepatocellular carcinoma
OS: Osteosarcoma
IL-6: Interleukin-6
DCS: Dendritic cells
RTP4: Receptor transporter protein 4
IHC: Immunohistochemistry
